# Emerging cross-talks between chronic kidney disease–mineral and bone disorder (CKD–MBD) and malnutrition–inflammation complex syndrome (MICS) in patients receiving dialysis

**DOI:** 10.1007/s10157-022-02216-x

**Published:** 2022-03-30

**Authors:** Shunsuke Yamada, Kazuhiko Tsuruya, Takanari Kitazono, Toshiaki Nakano

**Affiliations:** 1 Department of Medicine and Clinical Science, Graduate School of Medical Sciences, Kyushu University, 3-1-1 Maidashi, Higashi-Ku, Fukuoka, 8128582 Japan; 2 Department of Nephrology, Nara Medical University, Nara, Japan

**Keywords:** CKD–MBD, Hemodialysis, Inflammation, Malnutrition, MICS, PEW

## Abstract

Chronic kidney disease–mineral and bone disorder (CKD–MBD) is a systemic disorder that affects multiple organs and systems and increases the risk of morbidity and mortality in patients with CKD, especially those receiving dialysis therapy. CKD–MBD is highly prevalent in CKD patients, and its treatment is gaining attention from healthcare providers who manage these patients. Additional important pathologies often observed in CKD patients are chronic inflammation and malnutrition/protein-energy wasting (PEW). These two pathologies coexist to form a vicious cycle that accelerates the progression of various other pathologies in CKD patients. This concept is integrated into the term “malnutrition–inflammation–atherosclerosis syndrome” or “malnutrition–inflammation complex syndrome (MICS)”. Recent basic and clinical studies have shown that CKD–MBD directly induces inflammation as well as malnutrition/PEW. Indeed, higher circulating levels of inorganic phosphate, fibroblast growth factor 23, parathyroid hormone, and calciprotein particles, as markers for critical components and effectors of CKD–MBD, were shown to directly induce inflammatory responses, thereby leading to malnutrition/PEW, cardiovascular diseases, and clinically relevant complications. In this short review, we discuss the close interplay between CKD–MBD and MICS and emphasize the significance of simultaneous control of these two seemingly distinct pathologies in patients with CKD, especially those receiving dialysis therapy, for better management of the CKD/hemodialysis population.

**Supplementary Information:**

The online version contains supplementary material available at 10.1007/s10157-022-02216-x.

## Chronic kidney disease–mineral and bone disorder (CKD-MBD) as a systemic disorder that affects multiple organs and systems

Normal bone and mineral metabolism are critical biological processes required for the maintenance of the internal milieu and hard tissues that enable living animals and humans to move against gravity on Earth [1]. Among several organs involved in normal bone and mineral metabolism including kidney, bone, small intestine, soft tissues, and parathyroid glands, the kidneys play a central role [2]. The kidneys receive input through humoral mediators and neural networks, integrate the input, and alter the excretion and absorption of calcium and phosphate in the renal tubules to maintain a mineral balance in the whole body [3]. Accordingly, once kidney function declines, disruption of the mineral network gradually develops [4]. CKD–MBD is often recognized as abnormal values in serum biochemical parameters. As the chronic kidney disease (CKD) stage progresses, sequential changes in serum biochemical parameters arise. Briefly, circulating α-klotho levels decrease first, then serum fibroblast growth factor 23 (FGF23) levels increase, and serum calcitriol levels decrease in CKD stages 2 and 3, and serum parathyroid hormone (PTH) levels increase, followed by an increase in serum phosphate levels and a decrease in serum calcium levels [5].

Derangement of bone and mineral metabolism in patients with CKD was once regarded as a disease in the bone and parathyroid glands [6]. However, it is now regarded as a systemic disorder that affects a wide variety of organs and systems, including cardiovascular organs, and is referred to as CKD–MBD [7, 8]. Abnormal serum levels of phosphate, calcium, and PTH are associated with a heightened risk of morbidity and mortality in hemodialysis patients [9]. Several lines of evidence have confirmed that hyperphosphatemia is closely linked to increased incidence of cardiovascular events and death [10]. We further showed that hyperphosphatemia is associated with an increased risk of brain hemorrhage, sudden death, and intervention for peripheral arterial diseases in patients receiving maintenance hemodialysis [11–13]. Meanwhile, elevated circulating levels of PTH were reported to increase the incidence of bone fractures, cardiac hypertrophy, anemia, protein-energy wasting (PEW), and other organ dysfunctions in hemodialysis patients [14]. Furthermore, accumulating evidence has shown that increased serum FGF23 levels are associated with left ventricular hypertrophy, atrial fibrillation, vascular calcification (VC), infection, anemia, inflammation, and impaired immunity in hemodialysis patients [15]. Because serum levels of PTH and FGF23 increase in response to phosphate loading, lowering serum phosphate and reducing phosphate loading is of primary importance for the management of CKD–MBD in hemodialysis patients [16].

**Fig. 1:**
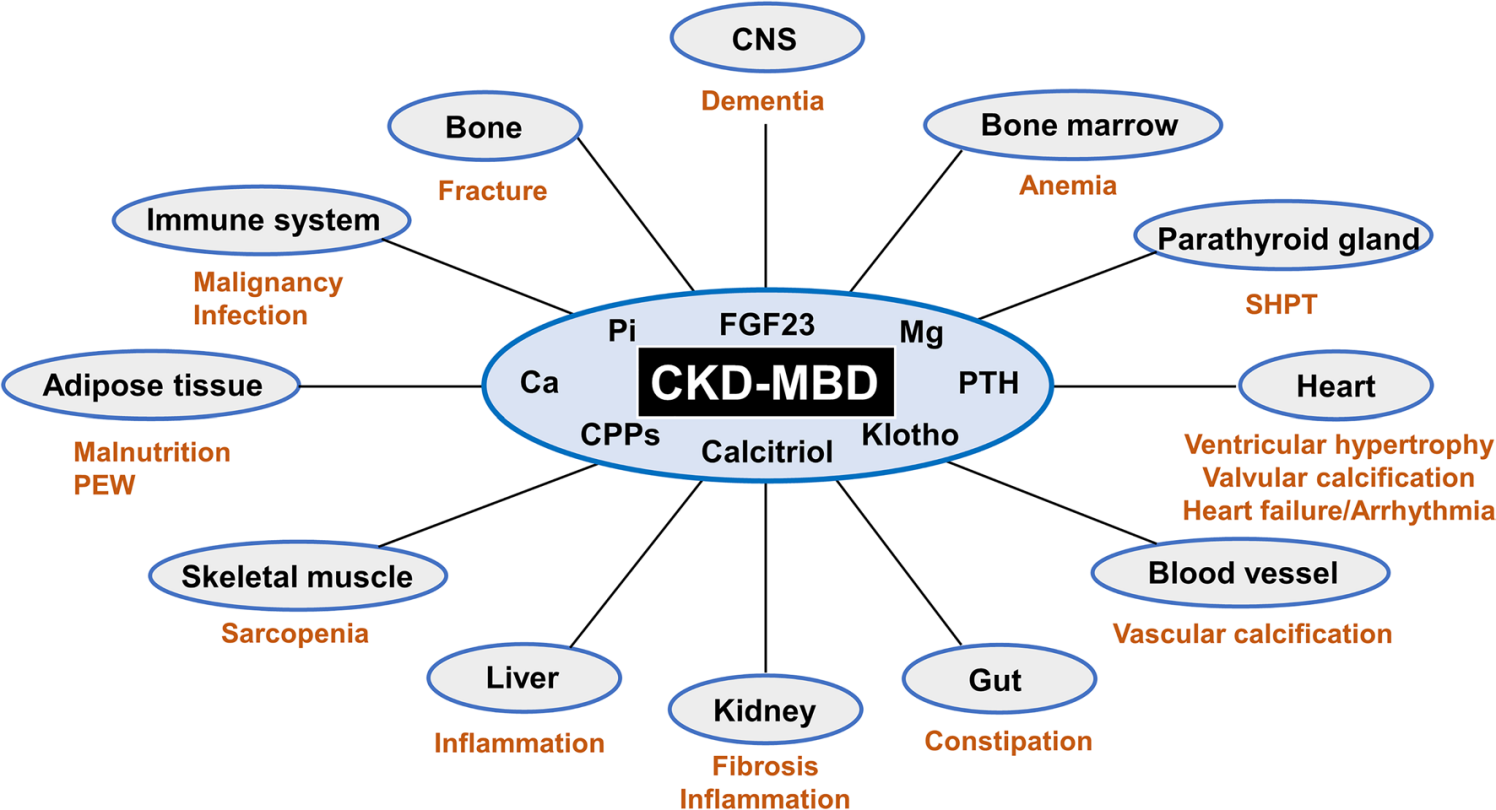
The expanding concept of CKD–MBD. CKD–MBD affects multiple organs and systems through dysregulated humoral mediators including Ca, Pi, CPPs, Mg, PTH, FGF23, calcitriol, and alpha-klotho. A variety of complications often observed in CKD presumed to be associated with CKD–MBD include dementia, anemia, SHPT, ventricular hypertrophy, valvular and vascular calcification, heart failure, arrhythmia, constipation, renal inflammation and fibrosis, liver inflammation, sarcopenia, malnutrition, malignancy, infection, and bone fractures. Abbreviations: *Ca* calcium, *CKD*–*MBD* chronic kidney disease-mineral and bone disorder, *CNS* central nervous system, *CPPs* calciprotein particles, *FGF23* fibroblast growth factor 23, *Mg* magnesium, *PEW* protein-energy wasting, *Pi* inorganic phosphate, *PTH* parathyroid hormone, *SHPT* secondary hyperparathyroidism

CKD–MBD is composed of laboratory test abnormalities, bone abnormalities, and VC, all of which can induce cardiovascular disease events, bone fractures, and other devastating complications, ultimately causing death in the CKD/dialysis population [7, 8]. Notably, through advances in our understanding of CKD–MBD pathogenesis, the concept of CKD–MBD continues to evolve and the boundaries of CKD–MBD are expanding to cover a wider range of diseases. Based on recent reports [14, 16–31], the complications considered to be associated with CKD–MBD include a variety of pathologies, such as dementia, anemia, secondary hyperparathyroidism, ventricular hypertrophy, valvular and vascular calcification, heart failure, arrhythmia, constipation, renal inflammation and fibrosis, hepatic inflammation, sarcopenia, malnutrition, malignancy, infection, and fractures (Fig. 1). It is of importance for medical practitioners engaged in the management of CKD/hemodialysis patients to be aware of the significance for appropriate management of CKD–MBD.

## The pathogenesis and mechanisms of VC in CKD

Among the three components of CKD–MBD, VC has gained increasing attention in recent years, and extensive research is being conducted to clarify its pathomechanisms in the uremic milieu [32]. VC is highly prevalent in the CKD population and was shown to be associated with increased cardiovascular morbidity and mortality [33, 34]. VC was once regarded as a passive and degenerative process of calcium-phosphate deposition in the vessel wall. However, it is now considered an actively regulated cellular process that resembles bone formation [35]. Over the last decade, basic research has revealed that VC is mediated by complex cellular mechanisms including transdifferentiation of vascular smooth muscle cells (VSMCs) into osteoblast-like cells, apoptosis of VSMCs, degradation of extracellular matrix, formation and release of calcifying matrix vesicles, and formation and maturation of calciprotein particles (CPPs) [36]. Among them, CPPs have been attracting the attention of researchers in the nephrology and CKD–MBD fields and are now assumed to be a critical mediator of VC [36, 37]. CPPs are loaded with calcium, inorganic phosphate, fetuin-A, and other proteins and increase in response to the phosphate and calcium burden, inducing inflammatory responses in leukocytes, monocytes, renal tubular cells, and VSMCs [37, 38]. When VSMCs are exposed to high CPP conditions, CPPs enter the intracellular space through scavenger receptor A or act on the cells through certain Toll-like receptors and induce intracellular calcium overload, resulting in mitochondrial dysfunction, apoptosis, altered autophagy, and calcification of the extracellular matrix [39, 40]. CPPs are now considered to be one of the strong drivers for uremic VC.

**Fig. 2:**
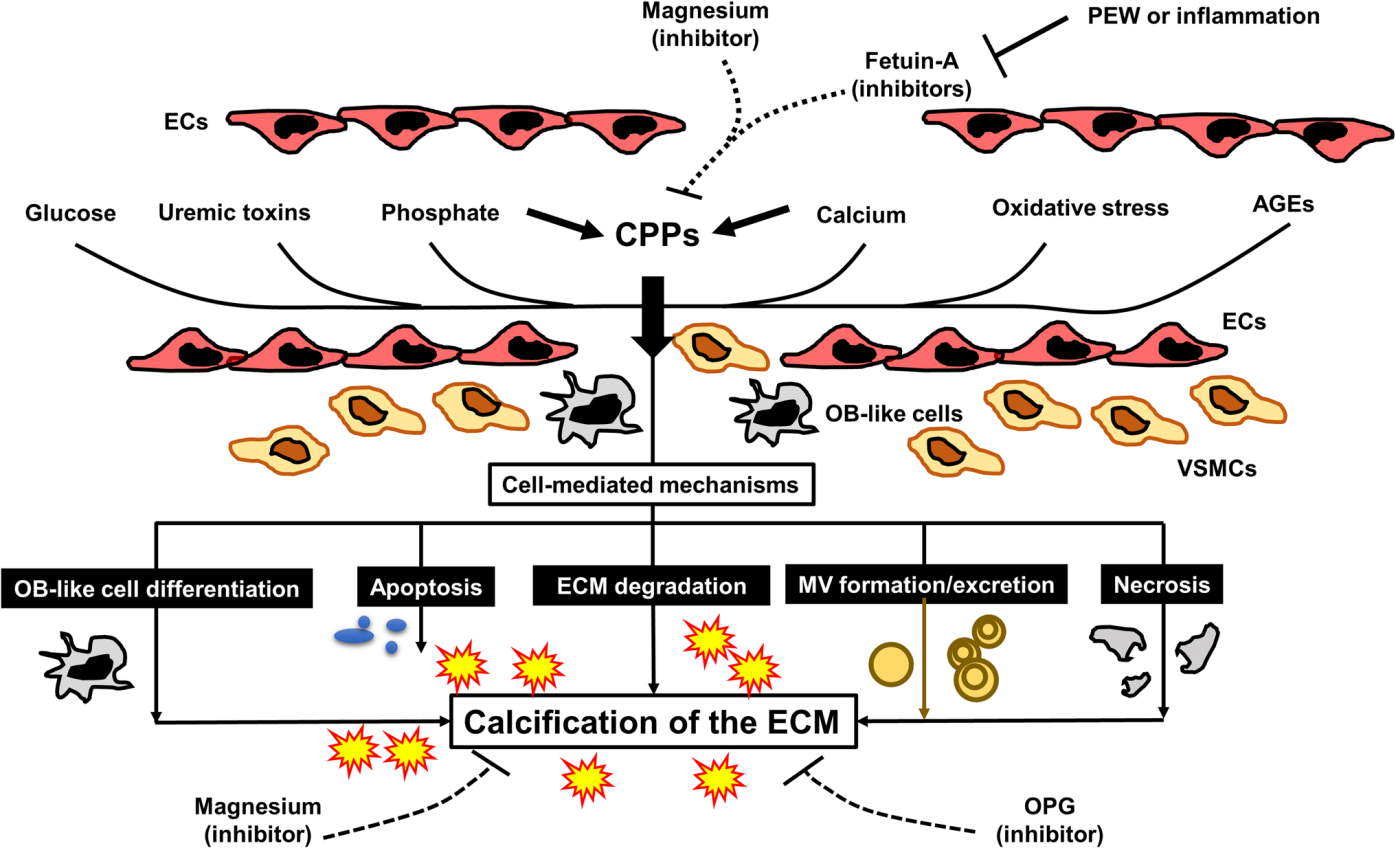
Mechanisms, inducers, and inhibitors of uremic VC. A variety of factors including CPPs have been shown to induce or accelerate the process of VC. VC is mediated by a series of cellular responses including transdifferentiation of VSMCs into OB-like cells, apoptosis of VSMCs, ECM degradation, MV formation, and cell death. These cellular responses synergistically promote calcification of the ECM in the medial layer of the blood vessel wall. Meanwhile, calcification inhibitors such as fetuin-A, magnesium, and OPG inhibit the calcification process by multiple steps. Abbreviations: *AGEs* advanced glycation end-products, *CPPs* calciprotein particles, *ECs* endothelial cells, *ECM* extracellular matrix, *MV* matrix vesicle, *OB* osteoblast, *OPG* osteoprotegerin, *VC* vascular calcification, *VSMCs* vascular smooth muscle cells

Another important viewpoint for VC is an imbalance between calcification inducers and inhibitors. In CKD, calcification inducers such as phosphate and calcium loading are accumulated, while calcification inhibitors such as fetuin-A, pyrophosphate, and magnesium in the circulation are decreased, thereby accelerating the VC process [41]. Unfortunately, treatment of VC remains challenging in clinical settings. However, basic studies have provided some clues toward VC prevention in the CKD population. In animal studies, phosphate loading dose-dependently induced VC and hyperphosphatemia in rodents with CKD, while dietary phosphate restriction or treatment with phosphate binders prevented or halted the progression of uremic VC [42]. Oxidative stress, which is highly augmented in the CKD/dialysis population, plays a pivotal role in VC pathogenesis, and treatment with antioxidants retarded VC progression in uremic rodents [43]. Meanwhile, magnesium ions, which were recently re-assessed as a potentially attractive therapeutic option in CKD, inhibited the formation and maturation of CPPs and prevented inflammation and VC in CKD [44]. Because VC is regarded as an irreversible biochemical phenomenon, it is very hard to regress once it has formed in the blood vessel wall, although some therapeutic interventions were reported to reverse VC [45, 46]. Accordingly, at present, prevention of VC is a reasonable and feasible strategy in the CKD/dialysis population. The pathomechanisms underlying VC are illustrated in Fig. 2.

## Malnutrition–inflammation–atherosclerosis (MIA) syndrome and malnutrition–inflammation complex syndrome (MICS) in patients with CKD

**Fig. 3:**
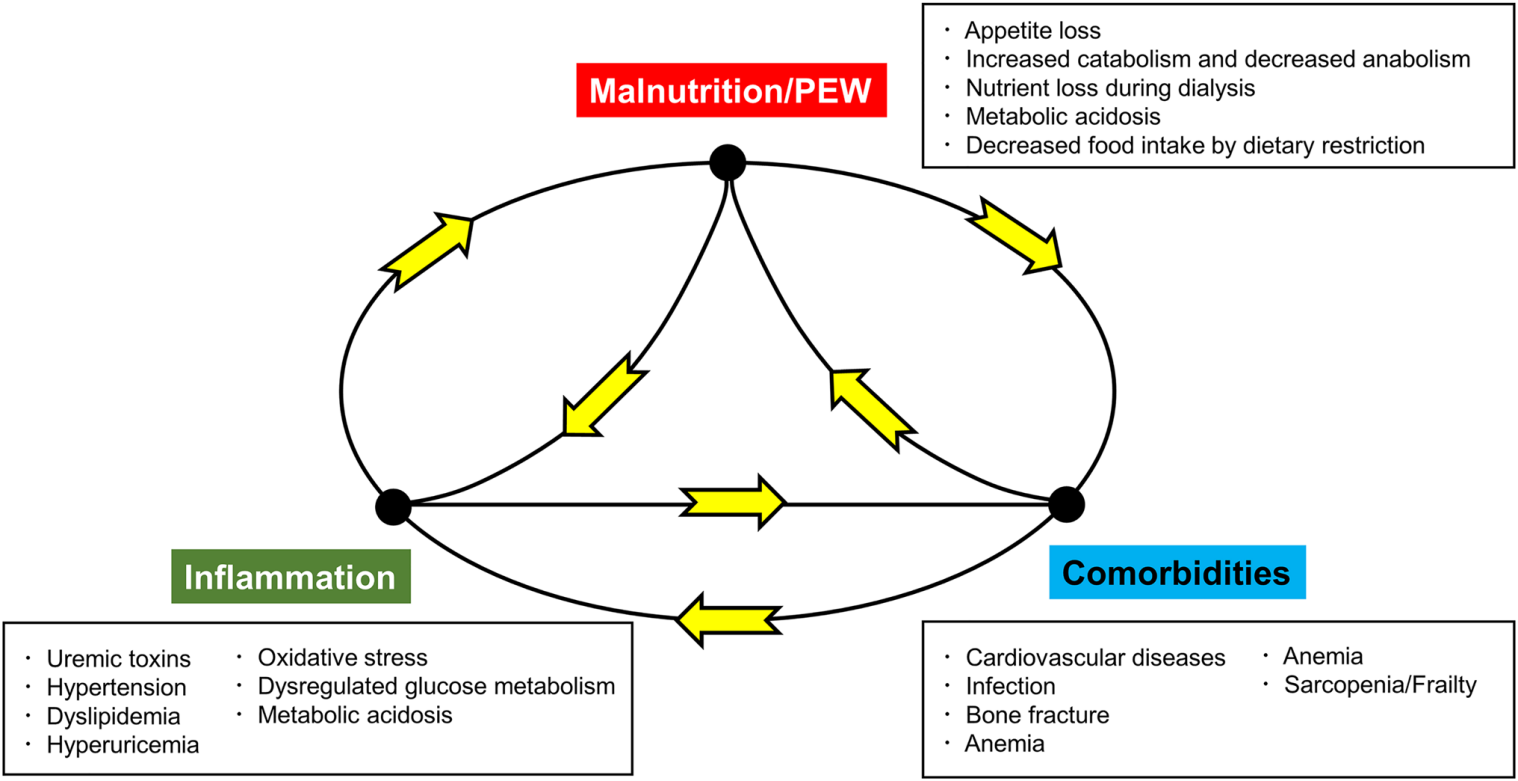
Schematic presentation of the interplay between malnutrition/PEW, inflammation, and comorbidities. There are cross-talks among inflammation, malnutrition/PEW, and complications in the CKD population. Inflammation and malnutrition/PEW cause a variety of complications and patients with comorbidity are prone to develop inflammation and malnutrition/PEW, forming a vicious cycle. Abbreviations: *CKD–MBD* chronic kidney disease–mineral and bone disorder, *MICS* malnutrition–inflammation complex/cachexia syndrome, *PEW* protein-energy wasting

Patients receiving hemodialysis are commonly complicated with inflammation and malnutrition [47]. Mounting evidence has shown that patients with malnutrition and inflammation have increased risk of morbidity and mortality [48]. These two interconnected pathologies create a vicious cycle and are tightly linked to the development and progression of cardiovascular diseases and other debilitating disorders [49]. Chronic inflammation in CKD was reported to be explained by the following pathologies observed in the uremic milieu: retention of uremic toxins such as indoxyl sulfate, p-cresol, and trimethylamine N-oxide, hypertension, dysregulation of glucose metabolism, dyslipidemia, hyperuricemia, and infection [50]. Regarding malnutrition, patients with CKD are likely to have low appetite due to azotemia, increased catabolism, persistent low-grade inflammation, decreased nutrient and calorie intakes due to dietary restriction, and nutrient loss during hemodialysis [51]. Basic studies have shown that malnutrition, inflammation, and oxidative stress in uremic patients synergistically contribute to cardiovascular system derangement [52]. To focus on the close links among malnutrition, inflammation, and atherosclerosis-related cardiovascular diseases or comorbidities, the terms “malnutrition–inflammation–atherosclerosis (MIA) syndrome” and “malnutrition–inflammation complex syndrome (MICS)” have been independently proposed by two research groups [53, 54]. The malnutrition–inflammation score (MIS) created for semi-quantitative assessment of MICS by Kalantar-Zadeh and colleagues was shown to be associated with the risk of mortality and other clinically important outcomes in patients receiving hemodialysis [55, 56]. The concepts for MICS and MIA syndrome are shown in Fig. 3.

Although the term “malnutrition” is often used to describe the poor nutritional status among CKD patients such as MICS and MIA syndrome patients, in recent years, it has been replaced by the term “protein-energy wasting (PEW)” in CKD patients, because the term “malnutrition” can indicate both undernutrition and overnutrition and is sometimes misleading [57]. Patients with CKD are often complicated with undernutrition, while overnutrition is rare. The term “PEW” was originally developed by the International Society for Renal Nutrition and Metabolism to describe the complex nutritional status in CKD patients, which cannot be explained by the general term “malnutrition”. Thus, PEW includes a broader scope of pathologies related to poor nutritional status in the uremic milieu: persistent inflammation, increased oxidative stress, appetite loss, hypercatabolism, acidemia, increased protein degradation with decreased protein synthesis, sarcopenia, intradialytic nutrients loss, and other important uremic-milieu-related pathologies that affect the nutritional status in CKD patients [57]. However, the term “malnutrition” may be more suitable in cases such as MIA syndrome and MICS, where the term “malnutrition” is incorporated in the definitions. Thus, in the present review paper, we have used these two terms interchangeably, and readers should note that both terms are used to indicate the same abnormal and complex nutritional status often observed in CKD patients. The term “malnutrition/PEW” is used hereafter.

## Assessment of nutritional and inflammatory status in CKD/hemodialysis patients

A variety of assessment tools for nutritional status and inflammation have been proposed and are available in clinical settings: serum levels of albumin, cholesterol, pre-albumin, transferrin, and creatinine, geriatric nutritional risk index, creatinine index, body mass index, normalized protein catabolic rate, handgrip test, and skeletal muscle mass measurement by bioelectrical impedance analysis or dual-energy X-ray absorptiometry [58, 59]. An ideal tool for assessment of the nutritional and inflammatory status should be objective, inexpensive, simple, intuitive, readily available at the bedside, and able to be performed without special machines. Unfortunately, no ideal assessment tools that fulfill all of these properties have been developed to date. Nevertheless, although not ideal, some of the recently proposed assessment tools can be clinically useful and applied for the time being, because malnutrition/PEW and persistent inflammation are critical issues in the CKD/hemodialysis population [60, 61]. Some of the clinically available tools for the assessment of nutritional status and inflammation are listed in Table 1.

**Table 1:** Reliability and practicability of assessment tools for nutritional status and inflammation in CKD patients

		Reliability (multifaceted, objectivity, predictability)				
		Very low	Low	Moderate	High	Very high
Daily practicability Cost Simplicity Repeatability Invasiveness	Very low					MIS
	Low		TST measurement	SGA	Handgrip test LBM measurement DEXA BIA	
	Moderate				Multifaceted index NRI-JH PEW-S	
	High	Blood tests Total cholesterol Choline esterase NLR	Blood tests Albumin Creatinine Pre-albumin CRP TNF-alpha IL-6 nPCR	Combined index creatinine index GNRI %CGN		
	Very high	Body weight	BMI			

Abbreviations: *BIA* bioelectrical impedance analysis, *CGN* creatinine generation rate, *CRP* C-reactive protein, *DEXA* dual-energy X-ray absorptiometry, *GNRI* geriatric nutritional risk index, *IL-6* interleukin-6, *LBM* lean body mass, *NLR* blood neutrophil to lymphocyte ratio, *MIS* malnutrition–inflammation score, *nPCR* normalized protein catabolic rate, *NRI-JH* nutritional risk index for Japanese hemodialysis patients, *PEW-S* protein-energy wasting score, *SGA* subjective global assessment, *TST* triceps skin thickness, *TNF-α* tumor necrosis factor-alpha

The panel of the International Society of Renal Nutrition and Metabolism proposed four components of PEW for multifaceted assessment of nutritional status: (i) biochemical indicators such as albumin or pre-albumin; (ii) low body weight, reduced fat, or weight loss; (iii) decreased muscle mass; and (iv) low protein or energy intake [57]. We and others have shown that a low PEW score, which includes one of the definitions for each component, is associated with an elevated risk of all-cause mortality and can be used as an assessment tool [62, 63]. Another useful tool for assessment of nutritional status is the nutritional risk index for Japanese hemodialysis patients (NRI-JH), created using a large database of Japanese hemodialysis patients [64]. The Japanese Society for Dialysis Therapy has proposed the NRI-JH as a new assessment tool for hemodialysis patients. The score has four categories, comprising serum levels of albumin, total cholesterol, and creatinine and body mass index, and the total score ranges from 0 to 13. We recently validated the NRI-JH score as a good nutritional tool and confirmed that a higher NRI-JH score is associated with worse survival in Japanese patients receiving maintenance hemodialysis [65]. Although these composite assessment tools are useful, further studies are necessary to build a better assessment tool that can estimate the overall nutritional status in CKD patients, given that malnutrition and inflammation are life-threatening complications. Furthermore, the potential application of the NRI-JH score to other ethnic groups requires confirmation.

As a comprehensive assessment tool, we recently developed a novel score for the assessment of MICS using the dataset from the Q-Cohort Study by applying bootstrapping, regression analysis, and risk prediction models [66]. The parameters included in the scoring system are age, body mass index, and serum levels of albumin, creatinine, and C-reactive protein. The MICS scoring system and its calculation are shown in the original paper. When this scoring system was applied to 3030 patients undergoing hemodialysis, patients with a higher MICS score had a higher risk of bone fracture, cardiovascular events, and death. For users who want to assess the nutritional status of hemodialysis patients independently of age, the scoring system is also available without age (Supplementary Table S1). Because the original MICS score is a continuous variable with a wide range (0–350), a semi-quantitative and simpler scoring system may be more useful and practical for medical practitioners. Accordingly, we have provided a simpler version of the original MICS score in Table 2. The simplified score ranges from 0 to 12. Serum Cr levels and body mass index are stratified by sex. In a future study, we will validate whether our new MICS scoring system developed for hemodialysis patients can serve as a good tool to detect patients at increased risk of malnutrition, complications, and death in independent cohorts of patients undergoing maintenance hemodialysis.

**Table 2:** Simplified scoring system for evaluation of MICS in patients undergoing hemodialysis

Variables	Category		Subscore
	Male	Female	
1. *Age (years)*	< 65		0
	≥ 65, < 75		1
	≥ 75		3
2. *Serum albumin (g/dL)*			
	≥ 3.9		0
	≥ 3.4, < 3.9		2
	< 3.4		3
3. *Serum Cr (mg/dL)*			
	≥ 12	≥ 10	0
	≥ 10, < 12	≥ 8, < 10	2
	< 10	< 8	3
4. *Serum CRP (mg/dL)*	< 0.06		0
	≥ 0.06, < 0.5		1
	≥ 0.5		2
5. *BMI (kg/m*^*2*^*)*			
	≥ 19	≥ 18	0
	< 19	< 18	1
*Total score*			0–12

The simplified version of the MICS score is composed of five elements that each have cut-off values. The total score is the simple sum of the five subscores, and ranges from 0 to 12. Serum Cr levels and BMI are stratified by sex due to the apparently different distributions of these two parameters in hemodialysis patients. The detailed statistical methods for the process of the original MICS score creation are described in reference #51 cited in the main text

Abbreviations: *BMI* body mass index, *Cr* creatinine, *CRP* C-reactive protein, *MICS* malnutrition–inflammation complex syndrome

## CKD–MBD as a direct inducer of inflammatory response and malnutrition/protein-energy wasting (PEW)

**Fig. 4:**
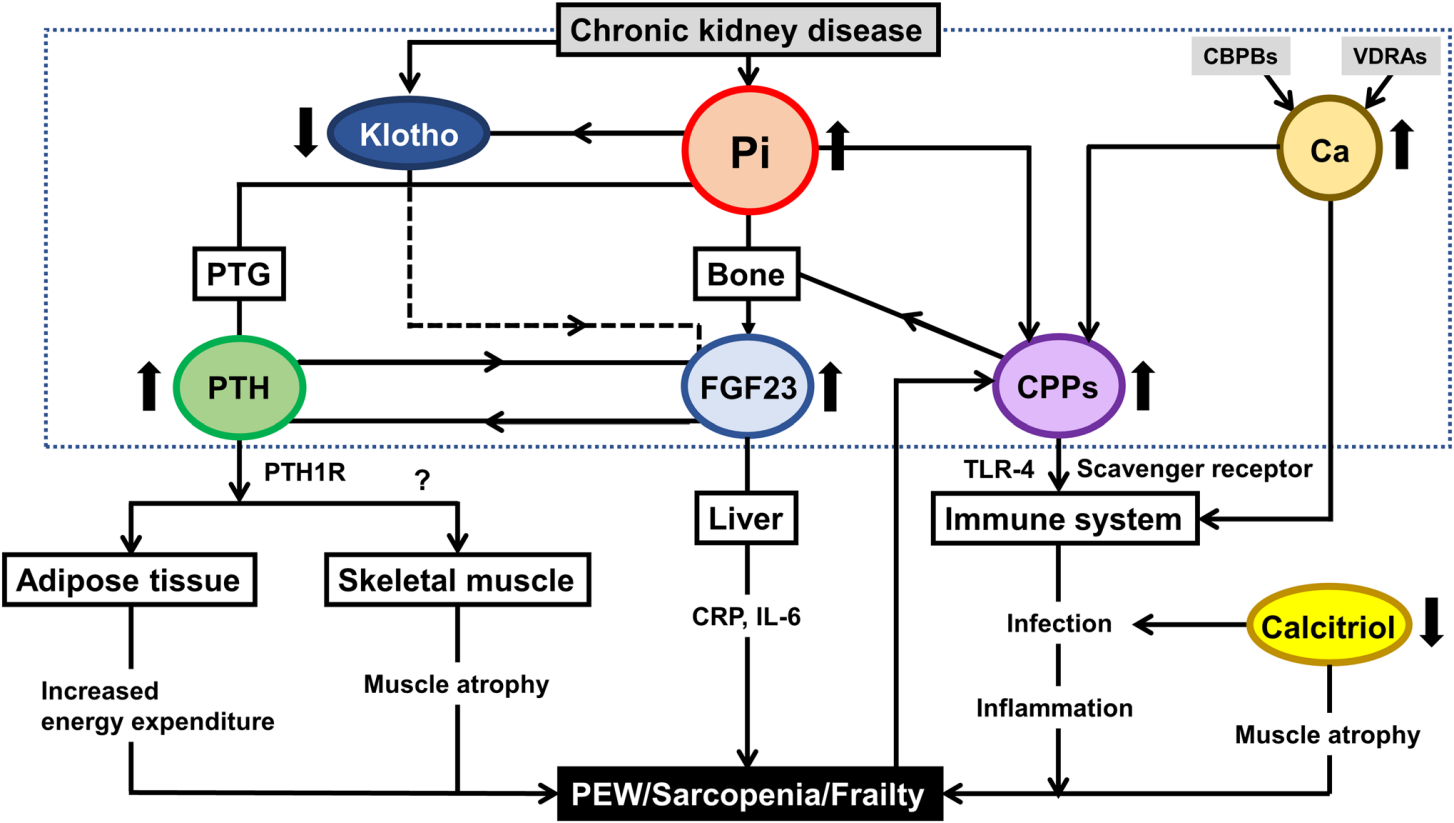
Schema showing the impact of CKD–MBD on PEW, sarcopenia, and frailty. In chronic kidney disease, phosphate accumulates in the body and serum levels of PTH, FGF23, and CPPs increase in the circulation. Klotho insufficiency or deficiency is also located in the upstream of the CKD–MBD sequence and increases FGF23 synthesis and secretion. In some patients treated with Ca-based phosphate binders and/or VDRAs, serum Ca levels are elevated. PTH acts on adipose cells and myocytes and increases energy expenditure and muscle atrophy. Increased Pi and Ca in the circulation lead to the formation and maturation of CPPs. FGF23 can directly induce inflammation via hepatocytes. CPPs, especially secondary CPPs, can impair the immune system and also induce inflammation via immune cells. Calcitriol deficiency in CKD is closely linked to impaired immunity, leading to an elevated risk of infection. Persistent infection causes inflammation, followed by malnutrition. Malnutrition then accelerates the maturation of CPPs, creating a vicious cycle. Collectively, these results strongly suggest that CKD–MBD causes malnutrition and inflammation, finally leading to sarcopenia and frailty. Notably, malnutrition and inflammation in turn aggravate CKD–MBD. Consequently, there is a need to simultaneously control CKD–MBD and MICS in patients receiving hemodialysis. Abbreviations: *CKD–MBD* chronic kidney disease-mineral and bone disorder, *Ca* calcium, *CBPBs* Ca-based phosphate binders, *CPPs* calciprotein particles, *CRP* C-reactive protein, *FGF23* fibroblast growth factor 23, *IL-6* interleukin-6, *PEW* protein-energy wasting, *Pi* inorganic phosphate, *PTG* parathyroid gland, *PTH* parathyroid hormone, *PTH1R* PTH receptor 1, *TLR-4* Toll-like receptor 4, *VDRAs* vitamin D receptor activators

Through advances in basic and clinical research during the last decade, a new and unexpected aspect of CKD–MBD has emerged. Specifically, CKD–MBD is deemed to directly cause an inflammatory response and malnutrition/PEW in CKD/hemodialysis patients. The pathogenesis linking CKD–MBD to inflammation and malnutrition/PEW is illustrated in Fig. 4.

Several lines of basic research have shown that phosphate loading increases oxidative stress in cultured VSMCs and that antioxidant treatment prevents VC development in rats and mice with uremia [43, 67]. We previously reported that dietary phosphate loading on adenine-fed rats induces dose-dependent decreases in serum albumin levels and body weight in adenine-induced CKD rats [68]. Furthermore, dietary phosphate loading dose-dependently elevated tumor necrosis factor-alpha (TNF-α) mRNA levels in the kidney, heart, and aorta of the adenine-induced CKD rats. We further reported that a very low protein diet enhances phosphate-induced VC in uremic rats by decreasing circulating fetuin-A levels and that administration of fetuin-A prevents phosphate-induced calcification of the extracellular matrix in cultured human VSMCs [69].

A recent cross-sectional study of patients receiving maintenance hemodialysis demonstrated that patients in the highest tertile group stratified by serum FGF23 levels had a higher odds ratio for increased serum inflammatory markers [70]. Meanwhile, a basic study elegantly showed that FGF23 directly acts on hepatocytes and accelerates the synthesis and section of C-reactive protein and interleukin-6 via fibroblast growth factor receptor 4 in an in vitro model of cultured HEPG2 cells [71]. Conversely, FGF23 synthesis is stimulated by nuclear factor kappa-light-chain enhancer of activated B cells (NF-κB). NF-κB up-regulates Orail-1, a calcium ion (Ca^2+^) channel activated by the Ca^2+^ sensor stromal interaction molecule 1 (STIM1) following Ca^2+^ depletion of intracellular Ca^2+^ stores. Consequently, Orai1/STIM1 affects store-operated Ca^2+^ entry (SOCE). Another basic study showed that synthesis of FGF23 in bone is regulated by AMP-activated kinase (AMPK), an energy sensor [72]. Specifically, AMPK-deficient mice had significantly lower serum FGF23 levels than wild-type mice. Meanwhile, AMPK downregulates Orail-1, a calcium-selective ion channel, and AMP synthesis is regulated by SOCE, which requires Orail-1. In a different basic study, rats fed a high-calorie diet had significantly higher serum FGF23 levels than rats fed a low-calorie diet [72]. Furthermore, rapamycin, an inhibitor of mammalian target of rapamycin (mTOR), reduced serum FGF23 levels in mice fed a high-calorie diet [73]. These results collectively suggest that synthesis of FGF23 is upregulated by an inflammatory response and also linked to energy metabolism, although the interplay between FGF23 and energy metabolism remains to be elucidated [74].

Regarding PTH, a basic study recently showed that persistent higher circulating PTH levels in CKD induce a phenotypic change of white adipose tissue to brown adipose tissue via PTH receptor 1, increase energy expenditure in mice, and also cause muscle atrophy by unknown mechanisms [75]. These data were confirmed by an observational study showing that hemodialysis patients with high serum PTH levels are likely to develop bodyweight loss [23]. These findings collectively suggest that uncontrolled secondary hyperparathyroidism (SHPT) makes a substantial contribution to PEW, frailty, and cachexia in patients with CKD, especially those on dialysis therapy.

CPPs, which are loaded with calcium, inorganic phosphate, fetuin-A, albumin, and other proteins, is now regarded as one of the decisive drivers for VC and inducers of the inflammatory response in various organs including the kidneys [76]. CPPs are classified into two forms: primary and secondary. Of these, secondary CPPs are considered to be more toxic than primary CPPs and to induce inflammation and calcification of the extracellular matrix [77]. Very recently, a laboratory test was devised to estimate the calcification propensity of patients using serum samples and is increasingly being used in pre-clinical studies [78]. The most popular test is named “T50”, representing the transition time required for one-half of the primary CPPs to be converted to secondary CPPs. Patients with shorter T50 are more likely to develop VC, while patients with longer T50 are less prone to develop VC. A cross-sectional study revealed that multiple clinical factors were associated with shorter T50 in patients receiving hemodialysis [78]. According to the report, hypercalcemia, hyperphosphatemia, and hypomagnesemia were associated with shorter T50, while hypoalbuminemia and low serum levels of fetuin-A and pyrophosphate were associated with shorter T50. Given that albumin and fetuin-A are both synthesized in the liver and categorized as negative acute-phase proteins, these results suggest that CKD–MBD, inflammation, and malnutrition/PEW cooperatively accelerate the formation and maturation of CPPs and that simultaneous control of these two pathologies is critically important to prevent chronic inflammation and progression of cardiovascular diseases including VC in CKD patients. At present, malnutrition/PEW and inflammation may be the key comorbidities to target for the prevention of CPP formation and subsequent progression of cardiovascular diseases in patients with CKD, especially those on dialysis therapy.

Recently, frailty has become a common term to describe increased vulnerability to health problems and preconditioned state for multiple comorbidities and death [79]. Frailty is generally defined as an aging-related syndrome of physiological decline in subjects with advancing age, and is evaluated by a series of physical phenotypes: unintentional weight loss, exhaustion, slowness, low activity level, and weakness. The prevalence of frailty in the CKD population is estimated to be higher than that in the general population [80]. Notably, several studies have found that CKD–MBD is associated with frailty [81–84]. Although the concept and pathogenesis of frailty partly overlap with those of MIA syndrome, MICS, sarcopenia, and malnutrition/PEW, it may be practical to consider CKD–MBD as a risk factor for frailty in the CKD population.

## The relationship between sarcopenia and CKD–MBD

Sarcopenia, defined as decreases in skeletal muscle mass and strength accompanied by disability in daily living, has become a major social issue in the current aging society [85, 86]. Notably, the prevalence of sarcopenia is higher in the CKD population than in the general population. Because sarcopenia is closely linked to decreased activity of daily living and quality of life and increased risk of morbidity and mortality, prevention of sarcopenia is of utmost importance in aged patients with CKD [87]. The pathogenesis of sarcopenia is complex and largely overlaps with that of malnutrition/PEW. Malnutrition/PEW is often accompanied by sarcopenia.

Recently, clinical studies have shown that sarcopenia is associated with disorders in the target organs for CKD–MBD: bone and cardiovascular system [88, 89]. We found that sarcopenia, as evidenced by reduced skeletal muscle mass, was associated with increased risk of disorders in the target organs for CKD–MBD in patients receiving maintenance hemodialysis using the dataset from the Q-Cohort Study [90–92]. As a surrogate marker for skeletal muscle mass, we employed the Cr index, which is calculated using age, sex, dialysis adequacy (Kt/V for urea), and serum creatinine level. Patients with a lower Cr index were found to have a lower skeletal muscle mass measured by a creatinine kinetic method and bioelectrical impedance analysis [90]. Notably, patients with a lower Cr index are at increased risk of bone fractures, cardiovascular disease events, and infection-related and all-cause death in patients receiving hemodialysis [90–92].

Reduced protein intake as a cause of malnutrition is also related to the target organs for CKD–MBD. Normalized protein catabolic rate (nPCR) was shown to be closely correlated with the amount of protein intake in stable hemodialysis patients and can be used as a surrogate marker for protein intake in clinical settings. Clinical studies showed that a lower nPCR level is associated with an increased risk of mortality in hemodialysis patients [93]. We recently showed that low or high nPCR levels were associated with an increased risk of bone fractures in patients receiving hemodialysis [94]. Our results suggest that decreased protein intake as a manifestation of malnutrition exerts a detrimental effect on bone fractures, a representative outcome of CKD–MBD, and that nutritional status should be regarded as a critical effect modifier in the management of CKD–MBD.

The links between sarcopenia and serum CKD–MBD markers should also be mentioned. Basic and clinical studies have shown that hyperparathyroidism, one of the common pathogeneses of CKD–MBD, converts white adipose tissue into brown adipose tissue and decreases skeletal muscle mass [75]. Vitamin D deficiency in CKD patients is closely linked to sarcopenia and skeletal muscle disorder [95]. Cross-sectional studies showed that hyperphosphatemia was associated with lower skeletal muscle mass and hand grip strength in patients with advanced CKD [96, 97]. Given that skeletal muscle acts as a large reservoir for inorganic phosphate, patients with sarcopenia are likely to have higher serum phosphate levels or hyperphosphatemia that can cause sarcopenia via inflammation or other mechanisms. Collectively, these data suggest that CKD–MBD is involved in the development and progression of sarcopenia in the CKD population.

## CKD–MBD and the links among bone fracture, sarcopenia, and VC in CKD

A variety of risk factors including malnutrition/PEW are involved in the development of bone fracture [98]. CKD–MBD may partially contribute to malnutrition/PEW, thereby increasing the risk for bone fracture. Accumulating evidence has revealed that CKD–MBD, aging, smoking, vitamin D deficiency, sex hormone deficiency, inflammation, increased oxidative stress, and dysregulated glucose metabolism are associated with decreased bone strength and fracture [99]. Importantly, bone fracture often occurs after falls in patients with decreased bone strength [100]. CKD patients have an increased risk of falls, partially influenced by sarcopenia in their lower extremities. Studies have shown that CKD–MBD evidenced by high serum levels of phosphate, calcium, CPPs, and PTH and vitamin D deficiency directly causes inflammation, followed by malnutrition/PEW and sarcopenia [38, 68, 71]. High circulating PTH levels increase the risk for bone fracture, cachexia, and malnutrition/PEW [14, 23, 75, 101]. In addition, bone produces osteokines and some of these osteokines including osteoprotegerin are protective against VC [102]. In turn, VC can cause ischemic osteopathy and decrease bone mineral density, thereby leading to an increased risk of bone fracture [103].

Regarding the link between sarcopenia and VC, direct involvement of sarcopenia with VC has been proposed. As we discussed in the previous section, serum CKD–MBD markers are closely associated with sarcopenia. Notably, recent studies have shown that myokines produced by skeletal muscle affect vascular health. Irisin may be one of these myokines and production of irisin is decreased in CKD patients. A basic study showed that decreased synthesis of irisin in the skeletal muscle aggravated atherosclerosis, while irisin administration prevented atherosclerosis progression [104]. Sarcopenia may promote VC via decreased bone volume and bone strength. Because mechanical stress driven by muscle contraction is essential for maintenance of bone mass, patients with sarcopenia generate less mechanical force to the bone, leading to increased sclerostin synthesis and section. Sclerostin negatively regulates bone volume by suppressing Wnt/beta-catenin signaling [105]. Accordingly, sarcopenic patients with higher circulating sclerostin levels are likely to suffer from decreased bone mass and release a large amount of CPPs into the circulation, resulting in progression of VC [106]. VC prevents blood flow increase in response to demand from the peripheral arteries and may cause skeletal muscle ischemia, thereby creating a vicious cycle between VC and sarcopenia. Further studies are necessary to completely delineate the complex interactions among sarcopenia, decreased bone strength, and VC and the involvement of CKD with the interactions among skeletal muscle, bone, and the cardiovascular system in CKD patients.

## A pharmacological approach to CKD–MBD

Treatments for CKD–MBD include phosphate unloading, maintenance of calcium balance, control of SHPT, maintenance and enhancement of bone strength, prevention of VC, cardiovascular diseases, and death, and improvement in quality of life [107]. In recent years, increasing numbers of therapeutic options have become available and novel drugs targeting CKD–MBD are currently under preliminary clinical trials. To date, phosphate binders, vitamin D receptor activators (VDRAs), calcimimetics, and some drugs for osteoporosis are the cornerstones for pharmacological treatment of CKD–MBD.

A variety of phosphate binders are available and can be used on a tailor-made basis [108]. Each drug has its advantages and disadvantages. In the selection of phosphate binders, several viewpoints should be considered. The threat of hypercalcemia as a strong driver of VC has tuned our treatment strategy to avoidance of calcium loading in the selection of phosphate binders [109]. Furthermore, to avoid polypharmacy associated with phosphate binder use, we are tempted to select strong phosphate binders to reduce the total pill number [110]. The tight links among iron deficiency, renal anemia, and CKD–MBD has opened the door to the use of iron-containing or iron-based phosphate binders, expecting iron supplementation and subsequent improvement in blood hemoglobin level, cardiovascular diseases, and survival [111]. Whether a phosphate binder can cause constipation may be another viewpoint to choose phosphate binders because constipation has emerged as a critical complication that can lead to comorbidities including cardiovascular diseases [26]. Notably, phosphate-lowering drugs with different pharmacological properties are under investigation. Preliminary data have shown that inhibitors of pan-phosphate transporters and inhibitors of sodium–hydrogen exchanger 3 are effective for reduction in serum phosphate levels [112, 113]. In the near future, better combinations of phosphate-lowering drugs will enable us to achieve stricter control of serum phosphate levels, thereby providing better outcomes for patients with CKD [114].

Calcimimetics have become a cornerstone therapeutic option in the treatment of SHPT [115]. Formulas for both oral and intravenous administration are available, including cinacalcet, evocalcet, etelcalcetide, and upacicalcet [116]. In contrast to VDRAs, calcimimetics generally lower serum levels of calcium, phosphate, FGF23, and CPPs. In a recent study that controlled serum PTH levels within the same range using maxacalcitol or etelcalcetide, T50 were significantly longer in the etelcalcetide group [117]. As represented by the results of the ADVANCE study and the EVOLVE trial, accumulating evidence has shown that calcimimetics provide better control of SHPT and reduce the risk of bone fractures, parathyroidectomy, VC, valve calcification, cardiovascular events, and death [118, 119]. Calcimimetics could be used as a primary treatment option for control of SHPT without inducing hypercalcemia and hyperphosphatemia in hemodialysis patients.

In the calcimimetic-centered era, the role of VDRAs in the treatment of SHPT is dramatically changing. The worldwide trend for SHPT treatment is directed toward combinations of calcimimetics with low-dose VDRAs [120]. Given that VDRAs are a useful treatment option to induce a positive calcium balance, coordination of the hypercalcemic and hyperphosphatemic actions of VDRAs with other pharmaceutical and non-pharmaceutical approaches is necessary. The putative organo-protective effects of VDRAs indicated in observational studies have not been confirmed in randomized clinical controlled trials [121]. However, there remains a possibility that the diverse favorable actions of VDRAs could be maximized in some subgroups, especially when VDRAs are used in combination with calcimimetics. Hence, further studies are mandatory to identify subgroups who receive beneficial effects of VDRAs to improve the overall outcome of hemodialysis patients.

## Interventions to cut the vicious cycle among CKD–MBD, malnutrition/PEW, and inflammation

It remains a subject of debate whether interventions for malnutrition/PEW and chronic inflammation improve the prognosis of hemodialysis patients. A variety of approaches have been proposed and some were reported to be effective for improving the nutritional status. For example, intradialytic nutrition and exercise were reported to improve nutritional status and prevent sarcopenia in hemodialysis patients [122, 123]. However, evidence to justify nutritional support is still lacking. Hence, we may need to think of a different approach to improve the nutritional status in hemodialysis patients.

Another important approach to prevent malnutrition and inflammation would be targeting of infection. Infection has been the second leading cause of death in hemodialysis patients and will soon be the leading cause of death in Japan [124]. The frequent causes of infection are pneumonia, vascular access infection, and catheter-related infection. Because infection is often accompanied by inflammation and malnutrition, it is of great importance to prevent infection and to start prompt treatment and eradicate the pathogens once patients develop an infection. As malnutrition leads to impaired immunity, infection and malnutrition create a vicious cycle [125]. A strategy to disrupt this vicious cycle is one of the critical steps for improving survival in the CKD/hemodialysis population.

Regarding treatment of CKD–MBD, an approach to hyperphosphatemia should be cautiously chosen. Although dietary phosphate restriction is a useful approach, it may decrease protein and energy intake, leading to malnutrition. Therefore, phosphate binder use may be a more practical approach to attain a normal serum phosphate level and maintain a good nutritional status [126]. We and others have shown that phosphate binders reduce the risk of infection-related and all-cause death by maintaining a good nutritional status in patients receiving maintenance hemodialysis [127, 128]. In line with these observational studies, another study showed that increasing protein intake increases serum albumin levels and improves the prognosis of hemodialysis patients [129]. Collectively, these results suggest that phosphate unloading by phosphate binder use would be a reasonable strategy to avoid malnutrition/PEW and infection and provide longevity to the hemodialysis population.

Treatment with VDRAs and calcimimetics for patients with SHPT has the potential to cut the vicious cycle between CKD–MBD and malnutrition/PEW. Administration of VDRAs can improve the altered function of immune cells in the uremic milieu, thereby protecting against infection and avoiding infection-related malnutrition/PEW and inflammation [130]. Calcimimetics lower the serum levels of PTH, calcium, phosphate, FGF23, and CPPs, all of which were shown to induce inflammation and oxidative stress [38, 68, 71, 131]. Moreover, reduction in serum PTH can prevent PTH-dependent energy expenditure, cachexia, and sarcopenia, thus lowering the risk of malnutrition/PEW [23, 75]. Further studies are necessary to confirm whether interventions for CKD–MBD can prevent the progression of malnutrition/PEW and inflammation and cut the vicious cycle in CKD patients.

Finally, interventions for malnutrition/PEW and inflammation could mitigate the impact of CKD–MBD on outcomes. In clinical studies, the impacts of hyperphosphatemia and hypercalcemia were suppressed in patients with good nutritional status versus patients with malnutrition/PEW [132, 133]. Inflammation was shown to increase FGF23 synthesis and release [134]. Taken together, these results suggest that interventions for CKD–MBD and malnutrition/PEW have bidirectional effects, leading to amelioration of these critical pathologies and improved survival among the CKD population.

## The concept of a blood vessel/heart–bone–skeletal muscle axis as a new therapeutic approach to CKD–MBD-related organ damage

**Fig. 5:**
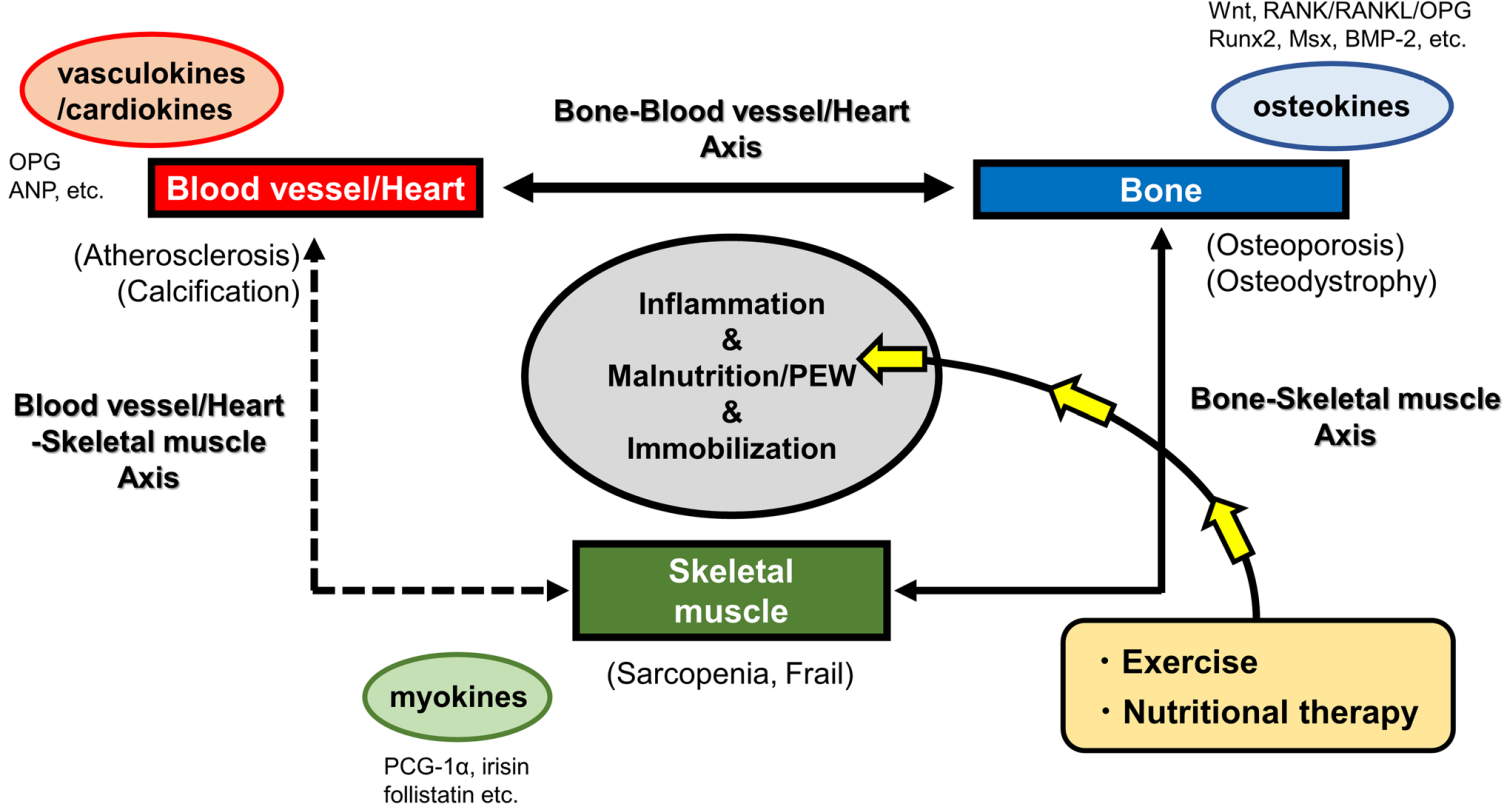
A novel concept of the blood vessel/Heart–Bone–Skeletal muscle axis in CKD. The blood vessels/heart, bone, and skeletal muscle have tight connections. These connections are termed the bone–blood vessel/heart axis, bone–skeletal muscle axis, or blood vessel/heart-skeletal muscle axis. Each organ synthesizes and secretes vasculokines, cardiokines, myokines, and osteokines, which affect all of the organs. Inflammation, malnutrition/PEW, and immobilization, which are highly common in the CKD population, could be critical inducers and accelerators of derangement in the blood vessel/heart–bone–skeletal muscle axis in patients with CKD. Therefore, exercise with appropriate nutritional therapy may disrupt the vicious cycle formed among the three axes and prevent cardiovascular diseases, osteoporosis/osteodystrophy, and sarcopenia/frailty in CKD patients. Abbreviations: *ANP* atrial natriuretic peptide, *BMI* body mass index, *BMP2* bone morphogenetic protein 2, *Cr* creatinine, *CRP* C-reactive protein, *MICS* malnutrition–inflammation complex syndrome, *Msx2* msh homeobox 2, *OPG* osteoprotegerin, *PEW* protein-energy wasting, *PCG-1α* peroxisome proliferator-activated receptor gamma coactivator-1 alpha, *RANK* receptor activator of nuclear factor kappa β, *RANKL* RANK ligand, *Runx2* runt-related transcription factor 2

To reduce the detrimental impact of CKD–MBD on survival in hemodialysis patients, different and novel approaches would be indispensable. Considering that bone, cardiovascular organs, and skeletal muscle are derived from the mesenchymal cell lineage, share potential plasticity, and develop and grow interdependently, it is reasonable to integrate these organs into a single system and make interventions for the entire system [135–137]. A bone–blood vessel/heart axis and a bone-skeletal muscle axis have been proposed, and recent research has revealed that these two axes share humoral mediators such as osteokines, myokines, cardiokines, and vasculokines and their receptors and affect one another through mechanical stress and mechanoreceptors. Interestingly, treatments for osteoporosis are also effective for VC, and treatments for sarcopenia increase bone volume [138, 139]. Meanwhile, treatments for atherosclerosis such as angiotensin receptor blockers improve bone strength [140]. These results partly suggest that risk factors are shared among atherosclerosis, osteoporosis, and sarcopenia and indicate that an integrated approach to the whole system may be effective for prevention of these disease conditions [141, 142]. One of the promising therapeutic approaches may be nutritional support with exercise, as described previously (143, 144). Indeed, decreased exercise generally stimulates bone resorption and decreases skeletal muscle mass and strength, while nutritional deficiency aggravates osteoporosis, sarcopenia, and vasculopathy. In future studies, it should be confirmed whether the impact of nutritional therapy with exercise can disrupt the vicious cycle of the bone–blood vessel-skeletal muscle axis and prevent deterioration in bone strength and progression of cardiovascular diseases and sarcopenia in the CKD/dialysis population. The concept of the bone–blood vessel/heart-skeletal muscle axis is illustrated in Fig. 5.

## Conclusions

CKD–MBD and MICS are highly prevalent complications in the CKD/dialysis population, have a close interaction with one another, and synergistically contribute to heightened risk of morbidity and mortality. Further research is necessary to determine whether simultaneous control of these two seemingly distinct pathologies can lessen the risk of morbidity and mortality, improve the quality of life and activity of daily living, and secure longevity in the CKD/dialysis population. Furthermore, a novel approach to CKD–MBD as a derangement in the blood vessel/heart–bone–skeletal muscle axis would be promising and effective for the prevention and treatment of CKD–MBD-related multiorgan dysfunction in this population.

## Supplementary Information

Below is the link to the electronic supplementary material.

Supplementary file1 (DOCX 53 KB)

## Abbreviations

AMPK: Adenosine-monophosphate-activated kinase
Ca^2^^+^: Calcium ion
CKD: Chronic kidney disease
CKD–MBD: Chronic kidney disease–mineral and bone disorder
CPPs: Calciprotein particles
Cr: Creatinine
FGF23: Fibroblast growth factor 23
MIA: Malnutrition–inflammation–atherosclerosis
MICS: Malnutrition–inflammation complex syndrome
NF-κB: Nuclear factor kappa-light-chain enhancer of activated B cells
NRI-JH: The nutritional risk index for Japanese hemodialysis patients
PEW: Protein-energy wasting
PTH: Parathyroid hormone
STMI1: Stromal interaction molecule 1
TNF-α: Tumor necrosis factor-alpha
VC: Vascular calcification
