# Supplementary material for: Emerging cross-talks between chronic kidney disease–mineral and bone disorder (CKD–MBD) and malnutrition–inflammation complex syndrome (MICS) in patients receiving dialysis
Source: Clin Exp Nephrol. 2022 Mar 30;26(7):613–29. doi: 10.1007/s10157-022-02216-x (PMC9203392; doi:10.1007/s10157-022-02216-x)
Supplement: Supplementary file 1 — Supplementary file1 (DOCX 53 KB) [file 10157_2022_2216_MOESM1_ESM.docx]

**SUPPLEMENTARY MATERIALS ONLINE**

**Emerging Cross-Talks Between Chronic Kidney Disease-Mineral and Bone Disorder (CKD-MBD) and Malnutrition-Inflammation Complex Syndrome (MICS) in Patients Receiving Dialysis**

Shunsuke Yamada, M.D., Ph.D.^1^, Kazuhiko Tsuruya, M.D., Ph.D.^2^, Takanari Kitazono, M.D., Ph.D.^1^, and Toshiaki Nakano, M.D., Ph.D.^1^

^1^Department of Medicine and Clinical Science, Graduate School of Medical Sciences, Kyushu University, Fukuoka, Japan

^2^Department of Nephrology, Nara Medical University, Nara, Japan

*Address for correspondence:*

Shunsuke Yamada, M.D., Ph.D.

Department of Medicine and Clinical Science, Graduate School of Medical Sciences, Kyushu University, 3-1-1 Maidashi, Higashi-Ku, Fukuoka 8128582, Japan.

Tel: +81-92-642-5843; Fax: +81-92-642-5846

E-mail: [yamada.shunsuke.944@m.kyushu-u.ac.jp](mailto:yamada.shunsuke.944@m.kyushu-u.ac.jp)

**SUPPLEMENTARY TABLE**

**Table S1. The scoring system for the MICS assessment with age excluded using multiple cut-off values.**

| **S-Cr (mg/dL)** | | | | **BUN (mg/dL)** | | **S-Alb (g/dL)** | | **S-CRP (mg/dL)** | | **BMI (kg/m^2^)** | |
| --- | --- | --- | --- | --- | --- | --- | --- | --- | --- | --- | --- |
| **Males** | | **Females** | |  |  |  |  |  |  |  |  |
| <2 | 100 | <2 | 86 | <10 | 0 | <2.0 | 69 | <2 | 0 | <15 | 19 |
| <4 | 94 | <4 | 85 | <20 | 1 | <2.5 | 63 | <4 | 2 | <20 | 17 |
| <6 | 88 | <6 | 84 | <30 | 2 | <3.0 | 56 | <6 | 5 | <25 | 14 |
| <8 | 82 | <8 | 83 | <40 | 3 | <3.5 | 50 | <8 | 7 | <30 | 11 |
| <10 | 75 | <10 | 81 | <50 | 4 | <4.0 | 44 | <10 | 9 | <35 | 8 |
| <12 | 68 | <12 | 78 | <60 | 5 | <4.5 | 38 | <12 | 11 | <40 | 6 |
| <14 | 56 | <14 | 72 | <70 | 6 | <5.0 | 31 | <14 | 14 | <45 | 3 |
| <16 | 42 | <16 | 62 | <80 | 7 | <5.5 | 25 | <16 | 16 | ≥45 | 0 |
| <18 | 28 | <18 | 53 | <90 | 8 | <6.0 | 19 | <18 | 18 |  |  |
| <20 | 14 | <20 | 44 | <100 | 9 | <6.5 | 13 | <20 | 20 |  |  |
| ≥20 | 0 | ≥20 | 34 | <110 | 10 | <7.0 | 6 | <22 | 23 |  |  |
|  |  |  |  | <120 | 11 | >=7.0 | 0 | <24 | 25 |  |  |
|  |  |  |  | ≥110 | 12 |  |  | ≥24 | 27 |  |  |

The scoring system for MICS assessment without age was created using the 3,030 patients receiving hemodialysis. The detailed data and main results are presented in the reference 66 (*Yamada S et al. Kidney Med 2022*) cited in the main text. The total scores for males and females range from 0 to 227 for men and 0 to 213, respectively. SCr are stratified by sex. The five parameters included in the scoring system were chosen by using Cox proportional hazard risk regression, risk prediction rule, and bootstrapping as described in the reference #66 in the main text.

Abbreviations: BMI, body mass index; BUN, blood urea nitrogen; MICS, malnutrition-inflammation complex syndrome; S-Alb, serum albumin; S-CRP, serum C-reactive protein; S-Cr, serum creatinine.
